# Systematic and biogeographic review of the Staphylinini rove beetles of Lord Howe Island with description of new species and taxonomic changes (Coleoptera, Staphylinidae)

**DOI:** 10.3897/zookeys.638.10883

**Published:** 2016-12-07

**Authors:** Josh Jenkins Shaw, Alexey Solodovnikov

**Affiliations:** 1 Natural History Museum of Denmark, Zoological Museum, Universitetsparken 15, DK-2100, Copenhagen, Denmark

**Keywords:** Staphylininae, Staphylinini, systematics, biogeography

## Abstract

Lord Howe is an oceanic and relatively young island situated in an area of complex geological and therefore biogeographical processes. The island boasts a large number of endemic species, including many beetles, however, few groups are in an adequate state of systematic knowledge for biogeographic investigation. Recent advances in the systematics of the hyper-diverse rove beetle tribe Staphylinini on a global scale enable us to implement taxonomic changes for species from Lord Howe Island. With the improved systematics we are able to make more accurate biogeographic conclusions and set a framework for further more in-depth exploration of this unique island using rove beetles. Two new species are described: *Cheilocolpus olliffi* **sp. n.** and *Quediopsis howensis* **sp. n.** Taxonomic changes for the tribe are implemented resulting in the following new combinations: *Cheilocolpus castaneus* (Lea, 1925), **comb. n.**, *Cheilocolpus kentiae* (Lea, 1925), **comb. n.**, *Ctenandropus mirus* (Lea, 1925), **comb. n.**, and *Hesperus dolichoderes* (Lea, 1925), **comb. n.** With the updated state of knowledge, the Staphylinini fauna of Lord Howe Island appears to be mainly derived from lineages on mainland Australia.

## Introduction

Lord Howe Island (LHI) is the eroded remains of a 6.9-million-year old shield volcano situated about 600 km East of Australia (McDougall et al. 1981). At 11 km in length and 2.8 km wide at its widest point, the island is characterised by two peaks, Mount Gower (875 m) and Mount Lidgbird (777 m) (Woodroffe et al. 1995), which are both located in the southern half of the island. Given the islands’ volcanic origins, geographic position along the western edge of the Lord Howe Rise (a major component of the Melanesian Rift) and its exposure to environmental factors such as winds typically dominated by seasonal prevailing westerly winds and year-round south to southwesterly trade winds (Woodroffe et al. 1995), LHI presents an ideal opportunity to investigate island colonization and biogeography.

LHI exhibits an impressive level of endemism across many taxonomic groups. For example, almost half of invertebrates on LHI are endemic (Cassis et al. 2003). The island was declared a UNESCO World Heritage Site in 1982 partly because of its insular biota and significant proportion of endemic species. Despite these figures, it is apparent that much biodiversity from the island remains to be described. It is also important that proper sister-group relationships and respective taxonomic placement is established for many described but systematically poorly understood species.

As a big and diverse insect order, beetles (Coleoptera) are one of the best groups to investigate evolutionary forces and resulting biogeographic patterns on LHI. According to Cassis et al. (2003) 60% of LHI beetles are endemic. Cassis et al. (2003) also suggested at least ten species of beetles to be extinct on LHI due to predation by introduced rodents.

Staphylinidae (rove beetles) is the biggest family of beetles, with around 60,000 species so far described (Solodovnikov et al. 2013). About 45 species of rove beetles are known from the LHI, plus a number of morphospecies identified to genus level (unpublished checklist maintained by C. Reid). Many rove beetles are generalised predators or saprophages and therefore they are not dependent on any particular environmental factor such as host plants for phytophagous beetles. Therefore, staphylinids are a particularly suitable model for studying evolution and biogeography of LHI. The poor state of taxonomic and phylogenetic knowledge of rove beetles, especially in Australasia, acts as an impediment for their use for biogeographic studies, and the rove beetle fauna of the LHI is no exception. Despite this, the rove beetle tribe Staphylinini, comprising over 5500 species globally (Brunke et al. 2016), have become much better understood phylogenetically and can now be used for biogeographic purposes as well, including studies focussing on the Australo-Pacific region (e.g. Solodovnikov and Brunke 2016).

Here, we review Staphylinini of LHI in the context of biogeography. Prior to our study, knowledge about Staphylinini of the LHI was very limited. Therefore, we fill this knowledge gap by critically reviewing taxonomy of all described species of Staphylinini of the LHI, and studying some newly collected material, especially for the subtribe Amblyopinina Seevers, 1944 which is a predominant lineage of that tribe in the Australo-Pacific region. This led to the discovery of new species and proper generic placements of some hitherto described species presented in this paper.

## Materials and methods

Material was examined as either traditionally point-mounted specimens or as disarticulated wet preparations in small petri dishes containing glycerin. Specimens were studied using a Leica MZ APO stereomicroscope. Genitalia are stored in glycerin in capsules under their respective specimens. Photographs were taken using a Leica MZ 16 A dissection microscope with a Leica DFC450C camera or a Canon EOS 7D combined with a Visionary Digital Imaging System and stacked using the Zerene Stacker Software. Photos were edited in Adobe Photoshop CS6. Drawings were digitally inked from photos in Adobe Illustrator CS6. The following measurements were taken using an ocular micrometer and are given in millimetres (mm). HL – Head Length (from apex of frons to neck constriction), HW – Head Width (maximal, including eyes), PL – Pronotum Length (along midline), PW – Pronotum Width (maximal), EL – Elytral Length (from acute humerus to most distal apical margin (best taken in lateral view)), EW – combined Width of both Elytra (maximal, with elytra closed along suture). Total body length was taken from the apex of the frons to apex of abdomen. Data labels on holotype and paratype specimens are repeated verbatim; label data from additional material is standardized (not verbatim). A forward slash (/) indicates separation of labels and a semi-colon (;) indicates separation of specimens in the ‘Material examined’ sections. To both new species we attach our ‘holotype’ (red) and ‘paratype’ (yellow) labels with all necessary information.

Specimens in the present study are deposited in the following collections:

AMS
 Australian Museum, Sydney (C. Reid) 


ANIC
 Australian National Insect Collection, Canberra (C. Lemann) 


BMNH
 Natural History Museum, London (R. Booth, M. Barclay) 


FMNH
 Field Musuem of Natural History, Chicago (A. Newton, M. Thayer, C. Maier) 


SAM
 South Australian Museum, Adelaide (P. Hudson) 


QM
 Queensland Museum, South Brisbane (G. Monteith, G. Thompson) 


ZMUN
 Zoological Museum, University of Oslo, Oslo (V. Gusarov) 


## Results

### Taxonomy

#### Subtribe Amblyopinina Seevers, 1944

##### Genus *Cheilocolpus* Solier, 1849

###### Cheilocolpus kentiae

Taxon classificationAnimaliaColeopteraStaphylinidae

(Lea, 1925) comb. n.

Figures 1 , 2C

Heterothops kentiae Lea, 1925

####### Material examined.

**Type material. *Paratypes***: All 20 paratypes kept in three institutions are mounted on 9 cards (pins) in groups from 1 to 5 specimens, with each pin having the same label printed on green or plain paper: ‘On Kentia. Lord Howe I. A.M. Lea’. Additionally, respective groups of specimens on each pin have the following labels: 2 females,’Heterothops kentiae Lea. Lea, Co-type[preprinted label with Lea’s handwriting] / Cotypes / Paratype [blue printed label] / K 188918 [printed white label]’; 2 females, ‘Co-type [printed label] / Paratype [pale blue printed label] / K56145 / Paratype [dark blue printed label] / K 188917 [printed white label]’; 1 male [mounted on its back] and 1 female, ‘Heterothops kentiae Lea. H288 Lea, Co-type [preprinted label with Lea’s handwriting] / Paratype [pale blue printed label] / A.H. Elston Collection [printed label] / K 188916’ (all six specimens on three pins from AMS); 1 male, 1 female, ‘Heterothops kentiae. Lea, Co-type [preprinted label with Lea’s handwriting] / Department of Zoology. Natural History Museum. University of Oslo. (ZMUN) [printed label] / Syntype. V.I. Gusarov rev. 2005 [two red printed labels]’ (ZMUN); 3 males, 2 females, ‘Summit of Mt. Gover, L.H.I. A.M. Lea / Heterothops kentiae Lea, Co-type [preprinted label with Lea’s handwriting] / Cotypus, Lea don. A. Lea [purple label in M. Bernhauer’s handwriting] / Chicago NHMus M. Bernhauer Collection [printed label]’; 1 male, 2 females, ‘Heterothops kentiae Lea, Co-type [preprinted label with Lea’s handwriting] / Cotypus, Lea don. A. Lea [purple label in M. Bernhauer’s handwriting] / Chicago NHMus M. Bernhauer Collection [printed label]’ (FMNH); 2 males on the same card, ‘c/3079 / Heterothops kentiae Lea, Co-type [preprinted label with Lea’s handwriting]’; 2 specimens mounted on two separate cards but on the same pin, ‘Lord Howe I., A.M. Lea / C 3199 / Heterothops kentiae Lea, Co-type’(QM).

**Additional material, all from Lord Howe Island, Australia.** 5 specimens: Stevens Reserve, rotted log and bark litter with fungi, 23.v.1980, S. + J. Peck; 2 specimens, Intermediate Hill, Big Creek, malaise trap through tall forest, 18-30.v.1980, S. + J. Peck; 1 specimen, Intermediate Hill, Big Creek, litter under carrion baits, 30.x.1980, S. + J. Peck; 7 specimens, Far Flats, thatch palm litter with nuts, 21.v.1989, S. + J. Peck (ANIC); 9 specimens: Mount Gower, 650-882 m [various collection dates] (AMS); 1 specimen: Mount Lidgbird, leaf litter of Bird’s Nest Fern Asplenium goudeyi 1.5 m off ground, 21.x.2001, Ian Hutton (AMS).

####### Diagnosis.

**Figure 1.**
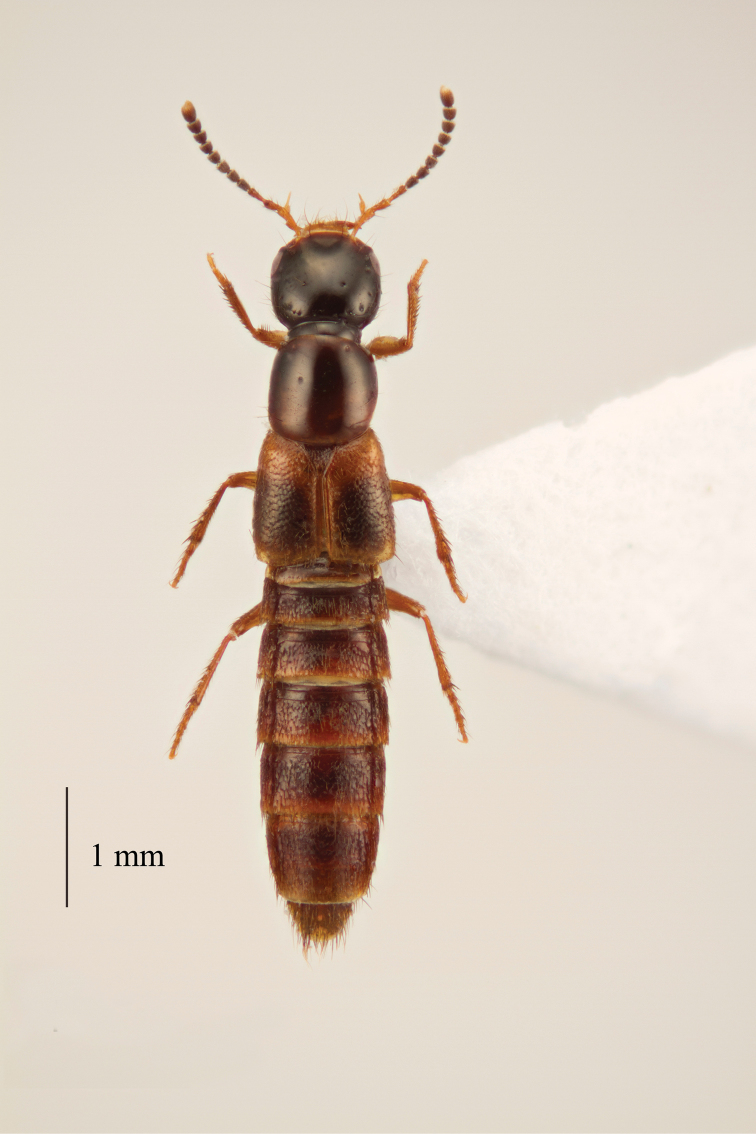
Habitus of *Cheilocolpus kentiae* (Lea, 1925).

**Figure 2.**
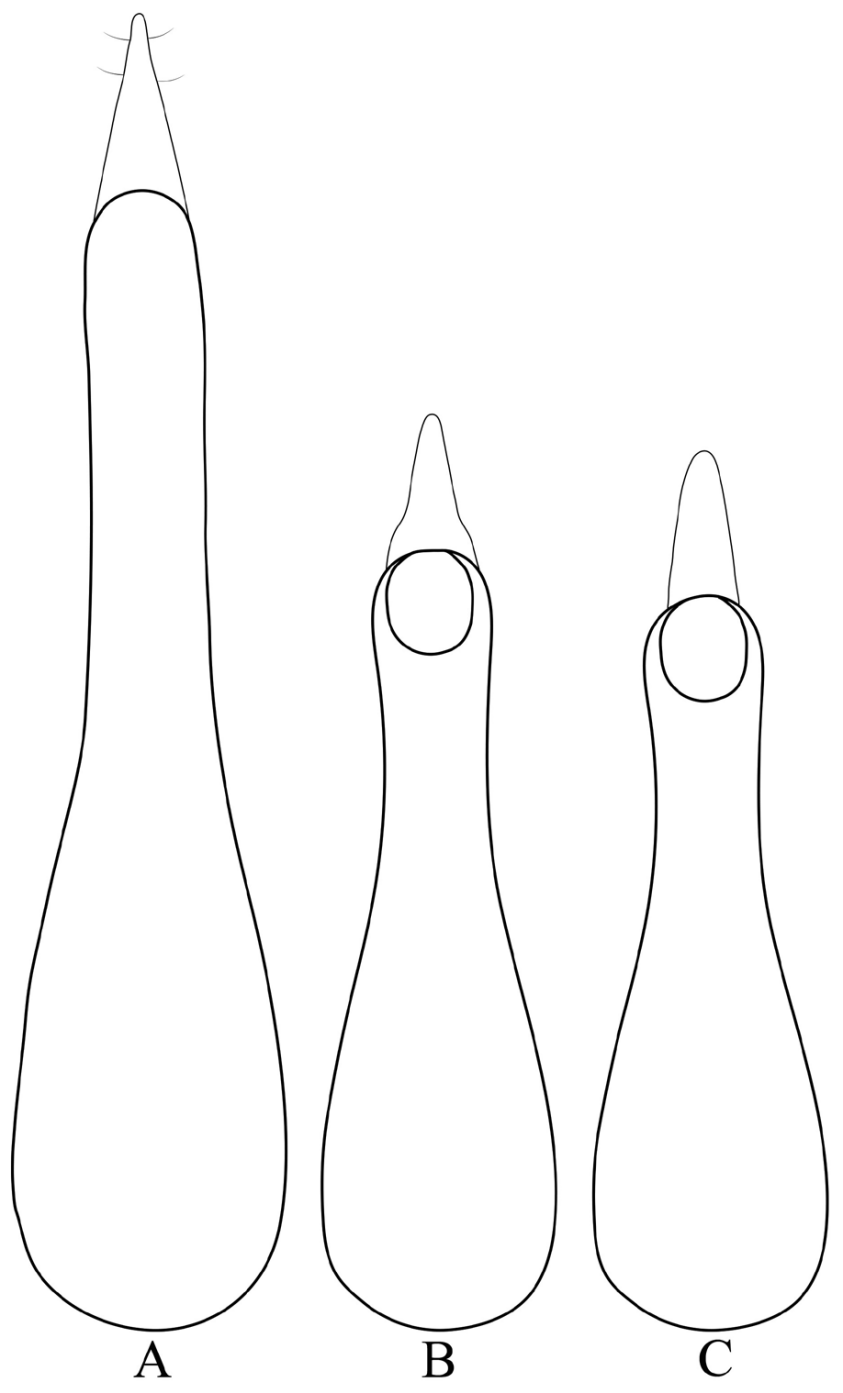
Aedeagi of Lord Howe Island *Cheilocolpus* (anti-parameral view). **A** *Cheilocolpus olliffi* sp. n. **B** *Cheilocolpus castaneus* (Lea, 1925) **C** *Cheilocolpus kentiae* (Lea, 1925).

Habitus as in Figure 1. Head as wide as, or wider than pronotum, black to dark brown with distinct microsculpture; infraorbital ridges short, far not reaching base of mandibles; postmandibular ridges well developed, extending towards gular sutures but not reaching them; postgena with scattered shallow punctures; eyes about a third of the size of the side of the head; antennomeres 1-3 yellow, 4-11 dark brown; distal antennomeres transverse; apical segment of labial and maxillary palpi aciculate. Pronotum dark brown with two punctures in each dorsal series and distinct microsculpture, hypomera without post-coxal process; elytra dark yellow, each elytron generally with posterior two thirds darkened; fully winged; legs yellow, tarsi with very long setae ventrally, protarsi with a few long white adhesive setae ventrally. Tergites III to V with anterior and posterior basal carinae; male sternite VIII without apical incision (unusual for most of Staphylininae); aedeagus with paramere; closely attached to, and apically protruding over, but median lobe and paramere still two distinctly separate entities, paramere apically rounded with several setae (Figure 2C).

####### Taxon discussion.

The original placement of *Cheilocolpus kentiae* in the genus *Heterothops* Stephens, 1829, like many other species of Australian Amblyopinina, was based on the aciculate last segment of maxillary palpi and resemblance in habitus. With such poorly justified generic identifications, *Heterothops* Stephens, 1829 was inflated to a genus of about 150 species from all over the world (Coiffait 1978; Smetana 1971, 1988; Herman 2001; Solodovnikov and Schomann 2009). *Heterothops* is based on the European species *Heterothops binotatus* (Gravenhorst, 1802) and its generic limits when including better known Holarctic species only (Smetana 1971; Coiffait 1978) are more clear. Holarctic *Heterothops* can be defined by the following character combination: long infraorbital ridges extending to base of mandibles; aciculate last segment of maxillary palps; pronotal hypomera without translucent post-coxal process; anterior tarsi dilated in both sexes; abdominal segments III-V with posterior basal carina connecting spiracles; and aedeagus with paramere entirely fused to median lobe so that both structures appear as one entity. Earlier this fusion was correctly recognized by Coiffait (1978) but misinterpreted as the complete loss of parameres by Smetana (1988).

With poorly studied global species diversity of ‘*Heterothops*’, it is not clear how far this character combination holds when Neotropical or Oriental species are considered (*Heterothops* is represented by one species in the Afrotropical region according to Solodovnikov and Schomann 2009). But, as far as the native Australian ‘*Heterothops*’ are concerned, it is clear that they are not congeneric with the Holarctic ones. Although the Australian and Holarctic species share some characters from the above mentioned diagnostic combination, the former do not have extended infraorbital ridges and their aedeagi have very distinct median lobe and paramere. At the same time, Australian ‘*Heterothops*’ share the same diagnostic character combination with the Chilean genus *Cheilocolpus* Solier, 1849, namely: infraorbital ridges poorly developed, short, never reaching the base of mandibles; apical segment of labial and maxillary palps aciculate, at base distinctly more narrow than apex of penultimate segment; pronotal hypomera without translucent post-coxal process; abdominal tergites III-V (or at least III) with anterior and posterior basal carinae, the latter connecting spiracles; paramere not fused with median lobe, distinct. Also, *Cheilocolpus* differ from *Heterothops* in habitus: the former (in dorsal view) have elongate more or less parallel-sided pronotum, while the latter have pronotum with sides narrowing anteriad.

The genus *Cheilocolpus* is based on *Cheilocolpus pyrostoma* (Solier, 1849) and, compared to other free living south temperate Amblyopinina, is relatively well monographed in a series of papers (Coiffait and Sáiz 1966; Sáiz 1971). Its limits with other related Neotropical genera such as *Rolla* Blackwelder, 1952 or *Philonthellus* Bernhauer, 1939 are not clear and must be investigated more elaborately. However, the listed shared character states and remarkable habitus similarity between the Australian ‘*Heterothops*’ species and smaller members of *Cheilocolpus* such as *Cheilocolpus angustatus* (Solier, 1849) from Chile, make it plausible to consider them congeneric. Such affinity is also biogeographically plausible in view of Gondwana-derived transantarctic connections between Australia and South America (e.g. Boger 2011). Even though we plan to move the main bulk of species of the Australian ‘*Heterothops*’ to *Cheilocolpus* in the course of a phylogeny-based generic revision of Amblyopinina (Jenkins Shaw & Solodovnikov, in prep.), here we already do so for *Heterothops kentiae* (and one other species, see below). Nomenclatural priority of the generic name *Cheilocolpus* over *Rolla* or *Philonthellus* also encourages this transfer now even though a separate genus status of these genera with respect to each other may be reconsidered in the future.

####### Note on the type material.

In the original description of *Heterothops kentiae*, Lea (1925) mentioned that he and his wife collected multiple specimens at Mt. Gower on fallen fronds and on wet parts of the *Kentia canterburyana* palm trees. He also indicated a specimen with the number ‘I.12690’ as a ‘type’. Based on the information in the original description, all specimens examined here are paratypes, many of which were apparently distributed by Lea among colleagues. Even though we did not examine the holotype (specimen with ‘I.12690’) that apparently is kept in Lea’s collection at the South Australian Museum in Adelaide, identity of the paratypes is unambigous.

###### Cheilocolpus castaneus

Taxon classificationAnimaliaColeopteraStaphylinidae

(Lea, 1925) comb. n.

Figure 2B

Heterothops castaneus Lea, 1925

####### Material examined.

**Type material. Holotype (male) and two paratypes (male and female), all from Lord Howe Island, Australia.** All three specimens mounted on the same card (1 pin). Holotype is far right male marked by Lea with letters ‘TY’ written on the card next to the specimen [here dissected with apical abdominal segments and aedeagus placed in the microvial with glycerin pinned under]. ‘I. 12691 Heterothops castaneus Lea. Lord Howe I. also slide [Lea’s handwritten label with the word ‘TYPE’ written in red ink along right margin] / castaneus. Lea, type. Lord Howe [small preprinted label with handwriting] / SAMA database No. 25-036194’ / Holotype (male, TY) and 2 Paratypes Heterothops castaneus Lea Jenkins Shaw & Solodovnikov rev. 2016 [red printed label] (SAM)’.

**Additional material, all from Mt. Gower summit at 850 m of elevation, Lord Howe Island, Australia.** 1 female, 850m, 27.ix.1978, T. Kingston, mossy forest’; 1 male, 9.xi.1979, G. B. Monteith / Q.M. BERLESATE No. 135. Volcanic soil, sieved litter. / Voucher Specimen 81-42 (QM)

####### Diagnosis.

Head about as wide as pronotum, brown, depigmented; infraorbital ridges short, far not reaching base of mandibles; postmandibular ridges well developed, extending towards gular sutures but not reaching them; postgena with several shallow punctures; eyes about a quarter or the size of the side of the head; antennomeres 1-3 slightly paler than 4-11; antennomeres 1-6 elongate; apical segment of labial and maxillary palpi aciculate. Pronotum brown, concolourous with head, with two punctures in each dorsal series and faint microsculpture, hypomera without post-coxal process; elytra brown, concolourous with head and pronotum; legs brown, concolourous with rest of body. Tergites III to V with anterior and posterior basal carinae; male sternite VIII without apical incision; aedeagus with paramere closely attached to, and apically protruding over, but paramere still distinct as structure separate from median lobe, apex of paramere rounded but more acute than *Cheilocolpus kentiae* (Figure 2B).

####### Taxon discussion.

*Heterothops castaneus* Lea, 1925 was described from Lord Howe Island where specimens were collected from leaf litter. Lea (1925) suggested its resemblance to species of *Calodera* (Aleocharinae) but also noted that it may be close to *Heterothops xantholinoides* MacLeay (1873) (=*Heterothops fauveli* Bernhauer & Schubert, 1916), a species from Australia which will also be transferred to the genus *Cheilocolpus* in due time (Jenkins Shaw & Solodovnikov, in prep). Here we transfer *Heterothops castaneus* to the genus *Cheilocolpus* for the same reasons as presented in ‘Taxon discussion’ for *Cheilocolpus kentiae*. Cassis et al. (2003) classified *Cheilocolpus castaneus* (there as *Heterothops castaneus*) as ‘Threatened Vulnerable’ and ‘Uncommon’.

####### Note on the type material.

In the original description of *Heterothops castaneus*, Lea (1925) mentions 6 specimens collected from fallen leaves. He also indicated a specimen with the number ‘I. 12691’ as the ‘type’. Here we examined three specimens from Lea’s collection in SAM, all mounted on the same card on one pin. Based on the information from the original description, among them a male marked by Lea with the letters ‘TY’ (for details see Material examined) is the holotype. All three specimens mounted on the same pin bear a handwritten label by Lea stating ‘I. 12691’ which reassures our correct interpretation of a holotype. The other two beetles (male and female) on the same card as the holotype are paratypes.

###### Cheilocolpus olliffi

Taxon classificationAnimaliaColeopteraStaphylinidae

Jenkins Shaw & Solodovnikov sp. n.

http://zoobank.org/CA56ADED-8397-4190-9A8D-F5169ACB35DD

Figures 2A , 3

####### Material examined.

**Type material. Holotype**: Male, point-mounted with apical abdominal segments in glycerin in capsule under specimen, with labels ‘AUSTRALIA: N.S.W., Lord Howe Island, 17-31.v.1980, S. + J. Peck / Intermediate Hill, Big Creek, 50’–200’, malaise trough, tall forest, 18-30.v.80 / Holotype *Cheilocolpus olliffi* Jenkins Shaw and Solodovnikov des. 2016’ (ANIC). **Paratypes** [all supplied with the labels ‘Paratype Cheilocolpus olliffi Jenkins Shaw and Solodovnikov des. 2016’: 3 males with locality labels same as holotype specimen. 5 paratypes with labels ‘AUSTRALIA: N.S.W., Lord Howe Island, 17–31.v.1980, S. + J. Peck / Intermediate Hill, 50’ pan traps, 19–30.v.1989’ (ANIC); 1 male with labels ‘NSW: Lord Howe Is., Mt Gower summit, c870m, 31°35'23"S. 159°04'21"E, 05Dec2000, C. Reid, Visitors book, mossy floor / K 188898 / H. sp2’ (AMS).

####### Description.

Measurements: HL 0.5–0.7; HW 0.5–0.6; PL 0.7–0.8; PW 0.5–0.7; EL 0.8–1; EW 0.8–1.1. Total body length 3.6–5.

**Figure 3.**
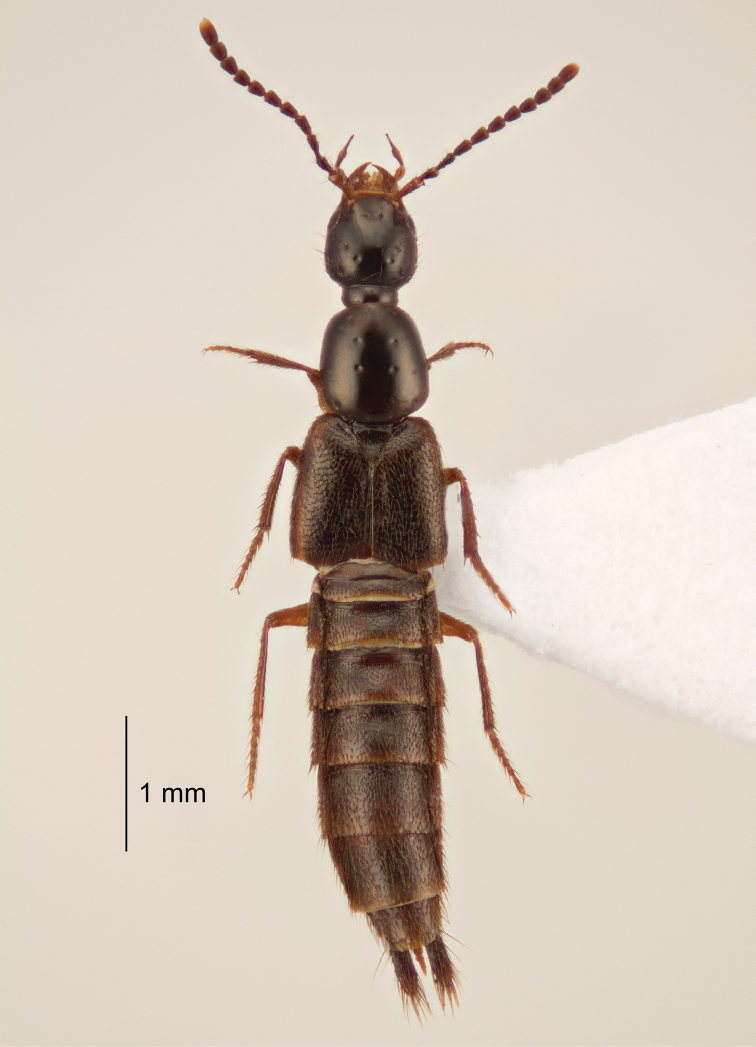
Habitus of *Cheilocolpus olliffi* Jenkins Shaw & Solodovnikov, sp. n.

Small, black to dark brown beetles. Habitus as in Figure 3.

Head capsule elongate more or less parallel-sided. Head surface with transverse microsculpture, on vertex with additional pair of punctures between anterior frontal punctures (sensu Smetana 1971), on temples sparsely pubescent. Length of eyes about one third of the side of the head. Nuchal ridge complete. Infraorbital ridge present but very short, far not reaching base of mandibles. Maxillary and labial palpi yellow, their apical segment aciculate. Labrum transverse, somewhat bilobed. Mandibles strongly produced. Dorsal mandibular ridge developed posteriorly. Gular sutures converging posteriad but not joining. Postmandibular ridge developed, directed towards gular sutures. All antennomeres elongate; first slightly paler than 2-11; 1-3 setose; 4-11 setose and with tomentose pubescence.

Surface of pronotum with transverse microsculpture and three pairs of punctures in dorsal series. Hypomeron strongly inflexed, and thus not visible in lateral view. Post-coxal process absent. Basisternum with pair of macrosetae.

Scutellum with only anterior scutellar ridge. Light brown, covered in setiferous punctures. Fully winged species, veins CuA and MP4 fused in one vein and vein MP3 present.

Legs yellow to orange with femora slightly darker than tibia and tarsi. Tarsal formula 5-5-5. Tarsi with sparsely distributed long setae ventrally.

Abdominal tergites III to V with anterior and posterior basal carinae the latter connecting spiracles.

Male. Sternite VIII without apical incision. Aedeagus with paramere closely attached to, and apically protruding over median lobe, but both still distinctly seen as two separate structures. Paramere apically acutely pointed with several small setae. In lateral view apical portion of paramere somewhat expanded. Apical tip of median lobe with narrow notch. Aedeagus of *Cheilocolpus olliffi* noticeably more elongated and about twice the length of the aedeagus of *Cheilocolpus kentiae* or *Cheilocolpus castaneus* (Figure 2A).

####### Diagnosis.

*Cheilocolpus olliffi* can be distinguished from other species of the genus from Australia based on the three pairs of punctures in the dorsal series of the pronotum and the pair of punctures on the frons between the eyes. Compared to the other LHI species, *Cheilocolpus kentiae* and *Cheilocolpus castaeneus* with respective antennomeres transverse, *Cheilocolpus olliffi* has antennomeres 6-11 elongate. All three LHI *Cheilocolpus* species have very distinctive habitus and aedeagi (Figure 2).

####### Etymology.

*Cheilocolpus olliffi* is named in recognition of Arthur Sidney Olliff (1865–1895), an English-born entomologist and taxonomist who made significant contributions to the understanding of LHI’s insect fauna, including recognising its affiliation with the mainland Australia. The species epithet is a noun in the genitive singular.

####### Taxon discussion.

The species is placed in the genus *Cheilocolpus* because it fully matches the diagnosis of the genus given in the Taxon discussion section under *Cheilocolpus kentiae*.

##### Genus *Ctenandropus* Cameron, 1926

###### Ctenandropus mirus

Taxon classificationAnimaliaColeopteraStaphylinidae

(Lea, 1925) comb. n.

####### Material examined.

**Type material. Holotype**: male [mounted on the same card with two female paratypes but marked with letters ‘TY’ by Lea], ‘mirus Lea, Type, Lord Howe I. / I.12703 Heterothops mirus Lea Lord Howe, also slide [Lea’s handwritten label]/SAMA Database No. 25-036156 (SAM); **Paratypes**: 2 females [mounted on the same card with the holotype], same labels as in holotype; 1 male, 2 females [mounted on the same card], ‘On Kentia Lord Howe I. A.M. Lea / co-type / Heterothops mirus Lea co-type, Lord Howe I.’ (SAM).

####### Taxon discussion.

*Heterothops mirus* Lea, 1925 was originally described from Lord Howe Island, based on specimens collected on *Kentia* palms. Lea (1925) noted its affiliation with *Heterothops magniceps* Bernhauer, 1920, in particular because of the unusually wide neck and extremely small eyes. The genus *Ctenandropus* was described by Cameron (1926) for a single species *Ctenandropus nigriceps* Cameron, 1926 that is presumably broadly distributed in Australia and Indonesia. Smetana (1988) complemented the detailed original description of *Ctenandropus* by additional diagnostic notes, redescribed *Ctenandropus nigriceps* and transferred *Heterothops magniceps* Lea, 1925 to that genus. *Ctenandropus* is one of the most easily recognizable genera of Amblyopinina due to its rather flat, small yellowish to brown body with very broad head having no neck constriction, and black combs in both sexes on the first mesotarsomere. The genus has wide distribution in the Oriental and Australo-Pacific regions and its species need revision which is not within the scope of this paper. Based on the study of type material of *Heterothops mirus* kept at the South Australian Museum which fully matches the diagnosis of *Ctenandropus*, we propose the new combination *Ctenandropus mirus* (Lea, 1925). Species level identification of the *Ctenandropus* from LHI must be further verified, as stated above.

##### Genus *Quediopsis* Fauvel, 1878

###### Quediopsis howensis

Taxon classificationAnimaliaColeopteraStaphylinidae

Jenkins Shaw & Solodovnikov sp. n.

http://zoobank.org/FE627261-1BA0-4057-9DCD-4BA77515CB5F

**Figure 4.**
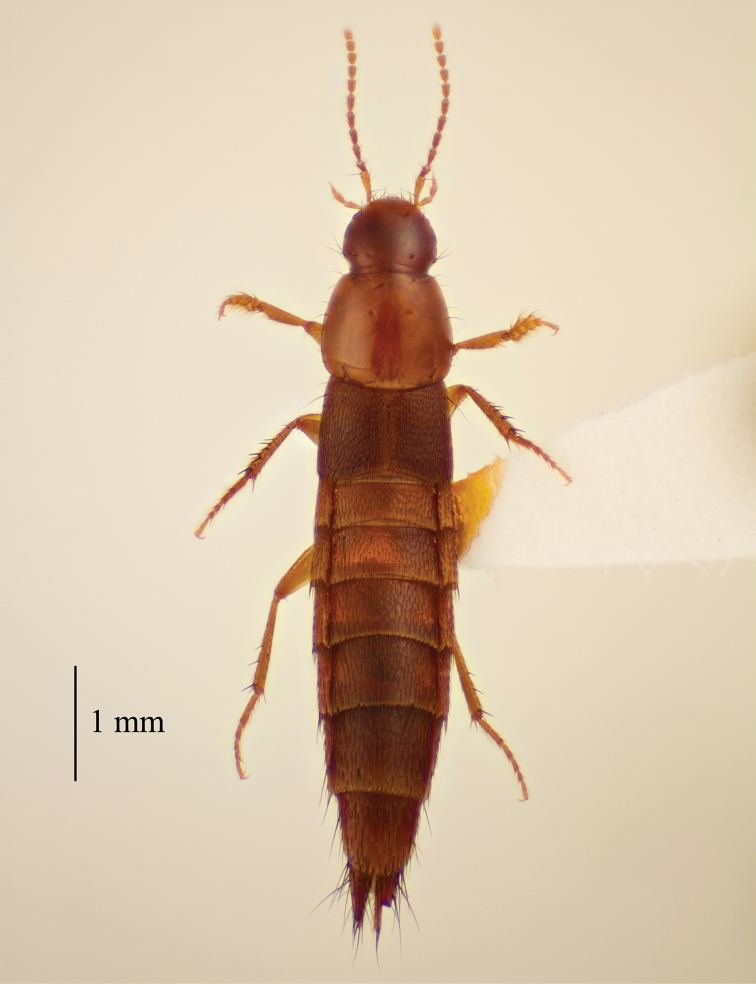
Habitus of *Quediopsis howensis* Jenkins Shaw & Solodovnikov, sp. n.

**Figure 5.**
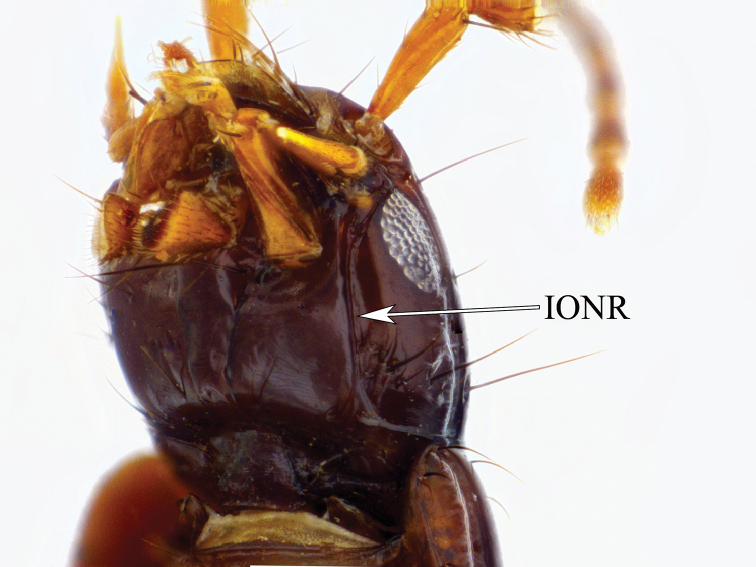
Lateroventral view of head of *Quediopsis howensis* Jenkins Shaw & Solodovnikov, sp. n. IONR = Infraorbital extension of nuchal ridge.

**Figure 6.**
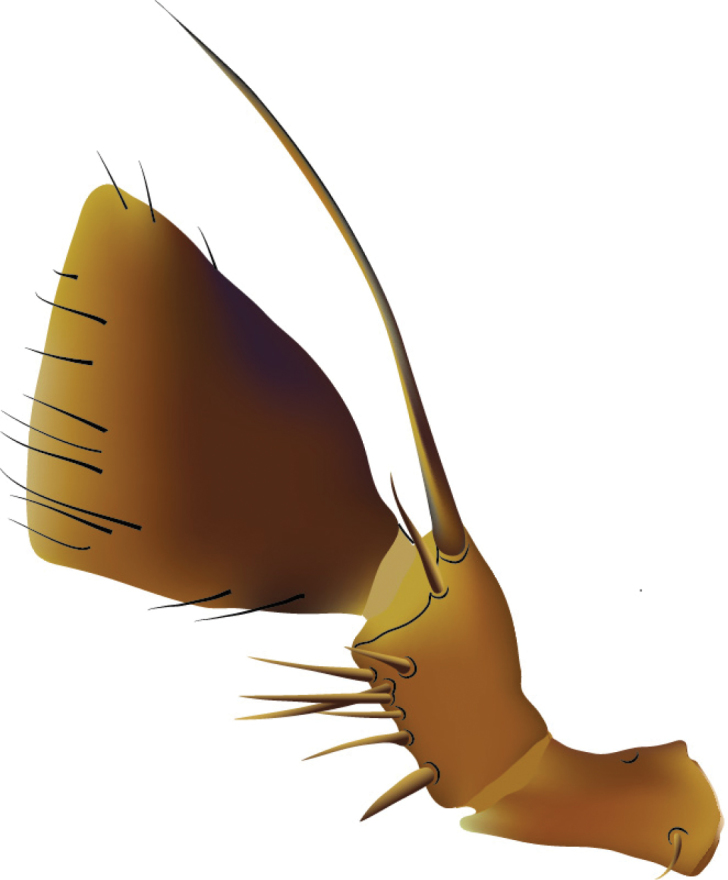
Labial palpi of *Quediopsis howensis* Jenkins Shaw & Solodovnikov, sp. n.

**Figure 7.**
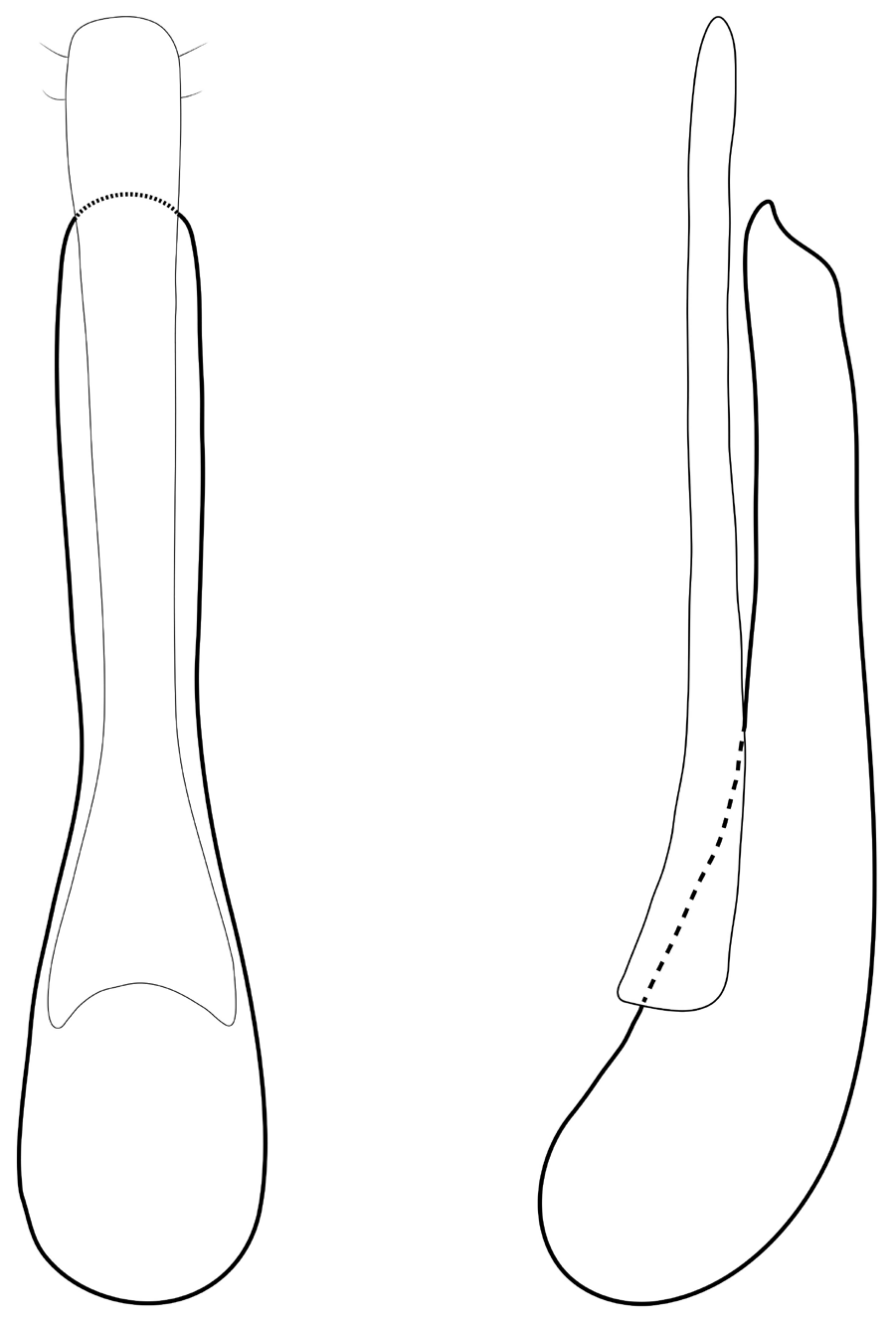
Aedeagus of *Quediopsis howensis* Jenkins Shaw & Solodovnikov, sp. n. Parameral view (left), lateral view (right). P = parameres, ML = median lobe.

Figures 4 , 5 , 6 , 7

####### Material examined.

**Type material. Holotype**: Male, point-mounted with apical abdominal segments in glycerin in capsule under specimen, with labels ‘Lord Howe Is: Mt Gower tk, 159°04'59"E 31°35'2"S, 730m N Velez 1-14 Nov 2004, Site G29 litter Zygonium, Dracophyllum forest / Australian Museum K403172’/ Holotype Quediopsis howensis Jenkins Shaw and Solodovnikov des. 2016’ (AMS). **Paratypes** [all supplied with the labels ‘Paratype Quediopsis howensis Jenkins Shaw and Solodovnikov des. 2016’: 1 female, ‘Lord Howe Is: Mt Gower tk, 159°04'49"E 31°35'9"S, 800m N Velez 1-12 Nov 2005, Site G32 litter Zygogy/Dracophyllum forest / Australian Museum, K403175’; 1 male, ‘Lord Howe Is: Mt Gower tk, 159°05'1"E 31°35'00"S, 690m N Velez, 10-17 May 2004, Site G27 litter, Hedy. canterburyana forest / Australian Museum K403173’; 1 female, ‘Lord Howe Is: Mt Lidgeb tk, 159°05'26"E 31°33'39"S, 360m N Velez, 1-14 April 2006, Site L11 litter, Drypetes/Cryptocarya / Australian Museum K403177’; 1 male, ‘NSW; On Soldiers Ck at NW junction, Lord Howe Is; -31:34:55; 159:5:9; 12/12/2003 to 22/12/2003; L. Meades, S. Lassau, G. Brown; RATSFC6-4P (pit / Australian Museum K403169’; 1 female, ‘NSW; Mt Gower, Lord Howe Island – Midway down ridge N of igloo; -31:35:5;159:4:35; 18-Jan-2002 to 31-Jan-2002; I. Hutton; ca. 819m; MG005 (pit trap) / K 188885 / Loan No. 1947, Australian Museum’; 1 male, ‘Lord Howe Is: Mt Gower tk, 159°05'10"E 31°34'50"S, 490m N Velez, 1-12 Nov 2004, Site G19 litter Dracophyllum/Metrosideros nervulosa scrub / Australian Museum K403174’; 1 female, ‘NSW; “Razorback”, Mt. Gower, Lord Howe Is; -31:35:30; 159:4:18; 28-Nov-2000; CBCR, Australian Museum; LHIS056L leaf litter ex Broad Close Sclerophyll Forest – Hedyscepe habitat / K188888 / Australian Museum, Loan No. 1947’; 1 female, ‘NSW; 1^st^ sites reached, next to Soldiers Ck, Lord Howe Is; -31:34:55; 159:5:9; 20-Apr-2004; L. Meades, S. Lassau, G, Brownl RATSCNF5-1L leaf litter ex: Lowland Mixed Forest litter / Australian Museum K403171’; 1 female, ‘Lord Howe Is: Mt Lidgbird tk 159°05'25"E 31°33'33"S, 260m N Velez 1-14 April 2006, Site L8 litter, Syzigium fullagarii forest / Australian Museum K403176’; 1 female, ‘NSW; Walking trail to Mt. Gower, at base of Scaly Bark Ridge, Lord Howe Is.; -31:34:47; 159:4:40; 02-Dec-2000 to 12-Dec-2000; CBCR Australian Museum; LHIS047/01 (pit trap) / K188882 / Loan No. 1947, Australian Museum’; 1 male, ‘NSW; Closest to 2^nd^ Tree, besides Golf Course, Lord Howe Is; -31:33:11; 159:5:1; 12/12/2003 to 22/12/2003; L. Meades, S. Lassau, G. Brown; RATGCFC4-2P (pit trap) / Australian Museum K402170’; 1 female, 1 male, ‘NSW; “Get Up Place”, trail to Mt. Gower, Lord Howe Is.; -31:34:58; 159:4:52; 02-Dec-2000; CBCR, Australian Museum; LHIS048L leaf litter ex Broad Closed Sclerophyll Scrub – Dracophyllum/Metrosideros habitat / K188883 / Loan No. 1947, Australian Museum’; 1 male, ‘NSW; 100m E from Soldiers Ck, closer to trail, Lord Howe Is; -31:34:55; 159:5:9; 12-Dec-2003; L. Meades, S. Lassau, G. Brown; RATSCFC8-3L leaf litter ex: Loweland Mixed forest Litter / Loan No. 1947, Australian Museum’; 1 male, ‘NSW; Western edge Golf Course, Lord Howe Is – Left side of clearing; -31:33:11; 159:5:1; 12/12/2003 to 22/12/2003; L. Meades, S. Lassau, G. Brown; RATGCNF1-2P (pit trap) / Loan No. 1947, Australian Museum’; 1 female, ‘NSW; Mt Gower, Lord Howe Island – Bottom of gully near igloo; -31:35:4; 159:4:31; 20/11/2001; I. Hutton, P. Flemons, C. Reid; MG003L leaf litter ex Bubbia – Dracophyllum / K188887 / Loan No. 1947, Australian Museum’; 1 female, ‘NSW; Mt Gower, Lord Howe Island – Midway down gull near igloo; -31:35:6; 159:4:32; 20/11/2011; I. Hutton, P. Flemons, C. Reid; MG002L leaf litter ex Bubbia – Dracophyllum / K188881 / Loan No. 1947, Australian Museum’ (AMS); 1 female, ‘AUSTRALIA: N.S.W. Lord Howe Island, 17-31.v.1980, S. + J. Peck / Mt. Gower, 850m, 26.v.80, rot wood w/fungi & moss, 12 L Ber’; 1 male, ‘AUSTRALIA: N.S.W. Lord Howe Island, 17–31.v.1980, S. + J. Peck / Stevens Reserve, 10’, 24.v.80, moist litter in limestone sink, 16 L Ber’; 1 male, ‘AUSTRALIA: N.S.W. Lord Howe Island, 17-31.v.1980, S. + J. Peck / Intermediate Hill, 300’, 18.v.1980, rotted bark w/fungi, tall forest, Ber 19 L / Quediopsis sp det. A.F. Newton 1987’ (ANIC); 1 male, ‘LORD HOWE ISLAND, Goat House Track, 11 Nov 1979, G.B. Monteith / Q.M. BERLESATE No. 138, Volcanic Soil, 250m, Pickard VegL Hb, Sieved litter’; 1 female, ‘LORD HOWE ISLAND, Mt Gower summit (NE), 9 Nov 1979, G.B. Monteith / Q.M. BERLESATE No. 134, Volcanic Soil, 850m, Pickard Veg: GMF, Sieved litter’; 1 female, ‘LORD HOWE ISLAND, Erskine Valley, north side, 22 Nov 1979, G.B. Monteith / Q.M. BERLSATE No. 160, Volcanic soil, 150m, Pickard Veg:CfLq, Sieved litter / VOUCHER SPECIMEN 81-40’ (QM).

####### Description.

Measurements: HL 0.7–0.9; HW 0.8–1.1; PL 1–1.2; PW 1.1–1.4; EL 0.8–1.1; EW 0.9–1.3. Total body length 5.9–7.6.

Medium sized, dark to light brown beetles. Habitus as in Figure 4.

Head brown-black in colour. Eyes about a third of the length of the head. Nuchal ridge continuing as ‘infraorbital extension’ to base of mandibles (Figure 5). Labrum transverse and bilobed. Mandibles with dorsal mandibular ridge indistinct. Mentum with alpha seta present (sensu Brunke and Solodovnikov 2013). Gula with weakly defined suture projecting posteriorly in the middle; gular sutures slightly narrowing medially. Labial palpi: apical segment securiform (expanded; or triangular in shape, Figure 6), covered in short setae; penultimate segment with long macrosetae extending over apical segment.

Antennomeres elongate, all of same colour. First antennomere slightly expanded apically, about as long as second and third combined.

Pronotum widest behind its middle at ca. posterior 2/3 of its length, slightly narrowed towards front angles; usually lighter than head in colour. Dorsal area with linear microsculpture and micropunctures visible at 40× magnification. Pronotal disk with two punctures in dorsal row: one very close to anterior margin of pronotum, and one on disc of pronotum before middle. Hypomeron inflexed and therefore not visible in lateral view, without post-coxal process. Basisternum with pair of black macrosetae and sometimes with other macrosetae positioned anterior to them.

Elytra usually the same colour as head, brown, sometimes with area around suture and scutellum rufous. Scutellum with only anterior scutellar ridge, setiferous in apical area. Sub-basal ridge present, not reaching humeral angles. Humeral angles with randomly positioned spine-like setae. Wings reduced, much shorter than elytra.

Legs concolourous. Tarsal formula 5-5-5. Both sexes with protarsi dilated and bearing dense white adhesive setae ventrally. Each tarsus with pair of empodial setae. Metacoxae almost parallel-sided along their entire length.

Abdomen usually the same colour as the pronotum. Tergites III and IV with anterior and posterior basal carinae, the latter connecting spiracles; tergite VII without apical seam of palisade fringe. Sternite III with basal transverse carina medially sharply pointed and forming an acute angle. Lateral tergal sclerites IX somewhat cylindrical, slightly flattened.

Male. Sternite VIII with apical emargination. Sternite IX with basal portion asymmetrical. Apical area of paramere somewhat spatulate with several short setae (Figure 7).

Female. Sternite VIII without emargination.

####### Diagnosis.

Among the described and all hitherto undescribed species of *Quediopsis* that we know from the material from throughout Australia, *Quediopsis howensis* may be separated based on the following combination of characters: apical segment of labial palpi extremely securiform; antennal segments concolourous; eyes slightly reduced; tergites III and IV with posterior basal carina connecting spiracles; apical margin of tergite VIII evenly rounded. Additionally, *Quediopsis howensis* is the only species in the genus with characteristic microphthalmous and poorly pigmented (brownish) body.

####### Etymology.

The species name refers to the fact that *Quediopsis howensis* seems to be a unique endemic representative of the genus on Lord Howe Island. It is an adjective derived from the latter part of the islands’ name.

####### Distribution.

The species is only known from Lord Howe Island and is probably endemic to the island.

####### Biology.

Nothing is known of the biology of the species, however label data associated with specimens indicates that is was collected in leaf litter of a variety of bush and tree species. The somewhat smaller eyes, distinctly depigmented body and fully reduced wings indicate that *Quediopsis howensis* may be adapted to life in deep layers of leaf litter.

####### Taxon discussion.

The genus *Quediopsis* Fauvel, 1878 was originally erected for two species from mainland Australia: *Quediopsis lugubris* Fauvel, 1878 and *Quediopsis abdominalis* Fauvel, 1878. Both species are very characteristic among all other Amblyopinina and share the following combination of characters that form the core of the diagnosis of the genus: apical segment of labial palpi securiform or wider than penultimate segment (Figure 6), covered in short setae; penultimate segment of labial palpi with long macro setae extending over apical segment; tergites III and IV and occasionally V with posterior basal carina in addition to anterior carina; nuchal ridge usually extending to base of mandibles (Figure 5). Subsequently, in the online database for Austral Staphylinoidea, Newton and Thayer (2005) proposed the transfer of *Quedius rubricollis* Fauvel, 1878 to *Quediopsis* (albeit without explanation). Even though the genus-level systematics of free living Amblyopinina is not developed and difficult as we have recently stated (Solodovnikov and Jenkins Shaw 2016), *Quediopsis* is a very clear cut and easy to recognise genus. Until now, no more species have been described in the genus, but based on the study of extensive material we estimate at least 6 new species of *Quediopsis* which still need to be described from mainland Australia. The genus seems to also present in Tasmania, New Zealand and New Caledonia but this will be investigated further in our forthcoming generic revision of the subtribe. The weakly defined sinuate suture on the gula characteristic for the new species from LHI is also found in at least two undescribed species of *Quediopsis* from mainland Australia.

#### Subtribe Philonthina, Kirby 1837

##### Genus *Cafius* Stephens, 1829

###### ‘Cafius’ gigas

Taxon classificationAnimaliaColeopteraStaphylinidae

Lea, 1929

Cafius gigas Lea, 1929

####### Taxon discussion.

This large, wingless rove beetle is only known from the type series collected from Mount Lidgbird. Although the exact location on Mt. Lidgbird or the habitat where specimens were collected is unknown, Lea (1929) states that they were ‘not taken from sea beaches’. *Cafius gigas* is now presumed to be extinct from Lord Howe Island due to predation by introduced rats early in the 20th century (Cassis et al. 2003; Priddel et al. 2003). The species is only represented by a handful of earlier collected specimens which are 130 years old. Based on our examination of morphology, *Cafius gigas* does not belong to *Cafius*, or even the broader ‘*Cafius*-complex’ sensu Jeon et al (2012). It seems to be a member of the Australian *Hesperus*-*Actinus*-*Leucitus* lineage of Philonthina where it may be sister or closely related to *Hesperus dolichoderes* (Lea, 1925), a peculiar species also endemic for Lord Howe Island (see below). The fact that *Cafius gigas* was collected from a non-coastal location (while all true *Cafius* are known exclusively from the sea shores) supports the morphology-based suggestion regarding its misplacement in that genus. A phylogenetic study where we are also attempting to extract DNA from *Cafius gigas*, and where the formal transfer of that species to the proper genus will be implemented is currently in preparation.

###### Cafius nauticus

Taxon classificationAnimaliaColeopteraStaphylinidae

(Fairmaire, 1849)

Philonthus nauticus Fairmaire, 1849

####### Taxon discussion.

*Cafius nauticus* was originally described in the genus *Philonthus* from Tahiti (Fairmaire 1849) and was later moved to *Cafius* by Fauvel (1874). *Cafius nauticus* was first reported from Australia by Fauvel (1903) and later Lea (1925), somewhat unclearly, suggested that *Cafius nauticus* was introduced to Lord Howe Island. In the phylogeny of Jeon et al. (2012), *Cafius nauticus* was based on a specimen from Australia and it was resolved as a sister group to the genus *Phucobius*, with *Cafius vestitus* Sharp, 1874 sister to the clade (*Cafius nauticus* + *Phucobius*). Undoubtedly *Cafius nauticus* belongs to the ‘*Cafius*-complex’ sensu Jeon et al. (2012) where, however, it was not assigned to any of the species groups but classified as *incertae sedis*. It is a wide-spread species known from Hawaii, Japan, China, Taiwan, Society Islands, Austral Islands, Samoa, Fiji, New Caledonia, Australia, Indonesia, Singapore, Sri Lanka, Madagascar, Seychelles, Mascarene Islands, Yemen, Eritrea, Djibouti, Somalia, Mauritius, Reunion, Rodriguez and, as an introduction, from Greece (Newton and Thayer 2005).

###### Cafius sabulosus

Taxon classificationAnimaliaColeopteraStaphylinidae

Fauvel 1877

####### Taxon discussion.

*Cafius sabulosus* was originally described from Sydney, Australia. In the phylogeny of Jeon et al. (2012), *Cafius sabulosus* was based on a specimen from Australia and formed a monophyletic group with *Cafius algophilus* Broun, 1894 from New Zealand. Similarly with *Cafius nauticus* (see above), in the revised classification of Jeon et al. (2012) *Cafius sabulosus* and *Cafius algophilus* were placed as *incertae sedis* within the ‘*Cafius*-complex’. According to Newton and Thayer (2005) *Cafius sabulosus* is also recorded from Queensland, New South Wales, Victoria, Tasmania, South Australia and Lord Howe Island.

##### Genus *Philonthus* Stephens, 1829

###### Philonthus antipodum

Taxon classificationAnimaliaColeopteraStaphylinidae

Fauvel, 1877

####### Taxon discussion.

*Philonthus antipodum* was originally described from Victoria and Queensland in Australia. Subsequently, Lea (1925) reported the species from New South Wales, South Australia, West Australia and Lord Howe Island. Without a comprehensive global phylogenetic study of the genus *Philonthus*, which according to Chani-Posse (2013) and Chani-Posse et al. (in press) is not monophyletic, currently it is impossible to assess sister-group relationships of this species. Preliminary study of the photos of a syntype in the BMNH suggests *Philonthus antipodum* may be closely related to or most likely a synonym of *Philonthus umbratilis* (Gravenhorst, 1802), a European species which is already known as adventive in New Zealand (Solodovnikov and Brunke 2016). Without examination of the rest of the type material of *Philonthus antipodum* at Fauvel’s collection in Brussels here we refrain from implementing a new synonymy. Also, material of that species from mainland Australia and Lord Howe Island, even though it superficially looks conspecific, remains to be carefully compared including genitalia dissections.

##### Genus *Hesperus* Fauvel, 1874

###### Hesperus dolichoderes

Taxon classificationAnimaliaColeopteraStaphylinidae

(Lea, 1925) comb. n.

Philonthus dolichoderes Lea, 1925

####### Taxon discussion.

This species was originally described in the genus *Philonthus* (Lea 1925) where it was still listed in the printed catalogue by Herman (2001). Recently Newton and Thayer (2005) proposed the new combination *Hesperus dolichoderes* (Lea, 1925) in their online database. They are credited for the taxonomic change that we here confirm and formally implement in printed publication. Despite *Philonthus* and *Hesperus* not being monophyletic (Chani-Posse 2013; Chani-Posse et al. in press), *Hesperus dolichoderes* is clearly a part of the *Hesperus*-*Actinus*- *Leucitus*-*Peucoglyphus* lineage of the Asian and Australo-Pacific region. Like many taxa in that lineage, *Hesperus dolichoderes* has a characteristically tube-like aedeagus with highly reduced paramere, a feature not characteristic to any lineage of a polyphyletic *Philonthus*. Until large scale revisions of both *Philonthus* and *Hesperus* complexes are carried out, *Philonthus dolichoderes* is moved to *Hesperus* even though this species is rather characteristic with unusually depigmented brownish body, brachyptery and smaller eyes presumably adaptations to island inhabitation. Usually *Hesperus* and related listed genera of that complex are well pigmented and often brightly coloured species with well developed eyes and hind wings. In spite of the peculiar habitus of *Hesperus dolichoderes*, its overall morphology does not conflict with the current broad and loose definition of *Hesperus*. As mentioned above, *Hesperus dolichoderes* and ‘Cafius’ gigas Lea, 1929 seem to be sister or at least closely related taxa, and both species will be treated in detail in a separate paper that is in preparation. The majority of *Hesperus dolichoderes* specimens studied by us were collected from Mount Gower by pitfalls traps. The species is endemic to LHI.

###### Hesperus pacificus

Taxon classificationAnimaliaColeopteraStaphylinidae

Olliff, 1887

####### Taxon discussion.

*Hesperus pacificus* Olliff, 1887 was originally described from LHI where it is endemic. In contrast to the above mentioned LHI endemic *Hesperus dolichoderes* and presumably closely related ‘Cafius’ gigas, *Hesperus pacificus* looks like a more typical species of the genus, i.e. more brightly coloured, with well developed wings.

#### Subtribe Staphylinina Latreille, 1802

##### Genus *Creophilus* Leach, 1819

###### Creophilus erythrocephalus

Taxon classificationAnimaliaColeopteraStaphylinidae

(Fabricius, 1775)

####### Taxon discussion.

*Creophilus erythrocephalus* is recorded from New Guinea, Australia, Lord Howe Island, New Caledonia, Tonga, Society Islands (French Polynesia), Hawaii, Easter Island, and finally Chile, where it is apparently introduced (Fauvel 1903; Clarke 2011). The species is found in open or disturbed habitats, often on dung and carrion; also can be attracted by light (Clarke 2011). It was first reported from LHI by Olliff (1889).

## Discussion

According to the most recent phylogeny of Staphylinini (Brunke et al. 2016), early evolution of this tribe displays a distinct biogeographic pattern. One of the most notable clades of Staphylinini that must have branched off early, the subtribe Amblyopinina, exhibits remarkable diversity in the temperate areas of the Southern Hemisphere and notable absence in their Northern Hemisphere temperate counterparts. In contrast, its sister clade that includes almost all other subtribes, is predominant in the temperate zone of the Northern Hemisphere and world (sub)tropics. This distinct biogeographic pattern was associated with early divergence within Staphylinini triggered by the break-up of Pangea into Laurasia and Gondwana (Brunke and Solodovnikov 2013; Brunke et al. 2016). Diversification of the predominately south temperate Amblyopinina took place on Gondwana-derived lands in isolation from the Laurasian landmasses where the majority of other Staphylinini have evolved. With continents gradually changing shape and forming connections, as well as through trans-oceanic or island hopping dispersal of some species, lineages of the southern and northern origin moved around to form modern complex mixed faunas on larger continents or island archipelagoes. Compared to large continents or island groups of complex history, more and continuously isolated landmasses like Australia, New Zealand or some smaller islands may display clearer biogeographic patterns that can be very useful in deciphering world biogeography. For example, a recent biogeographic review of Staphylinini from New Zealand revealed that 66% of New Zealands’ Staphylinini fauna are paleoendemic Gondwana-derived species of the subtribe Amblyopinina, while the rest of the fauna are either neoendemics or adventive species from the subtribes Staphylinina and Philonthina which (or their ancestors) reached New Zealand across sea gaps from the Laurasia-derived landmasses (Solodovnikov and Brunke 2016). That study of an insular New Zealand fauna in the context of recent phylogenetic hypotheses for Staphylinini went along with implementing some necessary taxonomic changes. Very poor taxonomic knowledge of the subtribe Amblyopinina, predominant in the Southern Hemisphere, remains a big obstacle on the way to more detailed biogeographic studies of New Zealand and other southern landmasses. The only recent species-level treatments of Amblyopinina concern the small genera *Mimosticus* (Brunke and Solodovnikov 2014) and *Myotyphlus* (Solodovnikov and Jenkins Shaw 2016).

The very limited fauna of Staphylinini on Lord Howe Island (LHI) with a number of unrevised or undescribed species of Amblyopinina seemed as an affordable next model to implement a biogeographic assessment similar to Solodovnikov and Brunke (2016). Besides, such an attempt was triggered by an extremely interesting biogeography and biology of the island (e.g. Buckley et al. 2009; Papadopulos et al. 2011). Prior to our taxonomic study of Staphylinini from LHI, it consisted of three endemic species of the globally distributed, poorly understood genus *Heterothops*, one endemic and two widely distributed species of *Cafius*, one Australian species of *Philonthus*, two endemic species of *Hesperus* and one species of *Creophilus* wide-spread in Australasian region.

Our study significantly changed the taxonomic and thus biogeographic composition of the LHI Staphylinini. About 40% of them are species of the Gondwana-derived subtribe Amblyopinina that are endemic on LHI: one species belonging to the Australo-Asian genus *Ctenandropus*; three (of which one is new to science) to the south temperate disjunctly distributed genus *Cheilocolpus*; and one to the mainly Australian genus *Quediopsis*. Based on our on-going study the most likely sister species to *Cheilocolpus* on LHI are to be found among unrevised species on the Australian mainland that are still placed in the genus *Heterothops*, such as *Heterothops ubiquitosus* Lea, 1925, *Heterothops nigrofrater* Lea, 1925 and *Heterothops laticeps* Fauvel, 1878. It is noteworthy that the *Sphingoquedius*-*Quediomimus* amblyopinine lineage which exhibits high diversity in Australia and New Zealand and also occurs on New Caledonia and Norfolk Island (example species: *Quedius luridipennis* MacLeay, 1871 and *Quediomimus hybridus* (Erichson, 1840)) is apparently absent from Lord Howe Island. The remaining 60% of Staphylinini on LHI are part of the Laurasia-derived so called ‘Staphylinini propria clade’ (Brunke et al. 2016). Of them, four species (*Cafius nauticus*, *Cafius sabulosus*, *Philonthus antipodum* and *Creophilus erythrocephalus*) are more or less wide-spread, associated with sea-shore based (*Cafius*) or broader decaying subtrates (*Philonthus* and *Creophilus*) and presumably colonised LHI from nearby Australia. Three species, *Hesperus pacificus*, *Hesperus dolichoderes* and the closely related species currently wrongly assigned to *Cafius*, *Cafius gigas*, are more interesting. Presumably a less specialized *Hesperus pacificus* colonised LHI in the same way as other species from the *Hesperus*-complex (Chani-Posse et al. in press) colonized Australia. The origin of highly derived *Hesperus dolichoderes* and ‘Cafius’ gigas maybe more complex and will be considered in a separate study.

## Supplementary Material

XML Treatment for Cheilocolpus kentiae

XML Treatment for Cheilocolpus castaneus

XML Treatment for Cheilocolpus olliffi

XML Treatment for Ctenandropus mirus

XML Treatment for Quediopsis howensis

XML Treatment for ‘Cafius’ gigas

XML Treatment for Cafius nauticus

XML Treatment for Cafius sabulosus

XML Treatment for Philonthus antipodum

XML Treatment for Hesperus dolichoderes

XML Treatment for Hesperus pacificus

XML Treatment for Creophilus erythrocephalus
